# Design Your Exam (DYE): A novel active learning technique to increase pharmacy student engagement in the learning process

**DOI:** 10.1016/j.jsps.2021.09.011

**Published:** 2021-09-30

**Authors:** Ahmad A. Shahba, Ibrahim Sales

**Affiliations:** a Department of Pharmaceutics, College of Pharmacy, King Saud University, Riyadh 11451, KSA; b Department of Clinical Pharmacy, College of Pharmacy, King Saud University, Riyadh 11451, KSA

**Keywords:** Active learning, Student-led, Assessment, Pharmacy education

## Abstract

**Background:**

Due to the COVID-19 pandemic, innovative e-learning solutions should be implemented to deliver knowledge to healthcare students remotely. Presently, there is a paucity of studies in the literature that have examined student-designed assessments in the classroom.

**Objectives:**

To examine the educational outcomes comparing the Design Your Exam (DYE) activity versus instructor-designed end-of-class (EOC) quizzes and explore student perceptions and preferences for each teaching modality.

**Methods:**

Lectures in the Industrial Pharmacy course were delivered to students by two different approaches: instructor-designed EOC assessments and student-designed DYE. The designed learning model was evaluated via an anonymous questionnaire for quality assurance and future course improvement.

**Results:**

Mean exam performance for content taught using the instructor-designed EOC quizzes and DYE activity were 74.4% and 71.9%, respectively (p = 0.092). Average student attendance for lectures taught using instructor-designed EOC quizzes and the DYE activity were 77.6% and 72.1%, respectively (p = 0.524). A post-course survey showed that 72.2% preferred the instructor-designed EOC, 5.6% preferred DYE activity, and 16.7% preferred a combination of the activities. Respondents reported that the EOC quizzes helped them to understand the lecture material and kept them focused during the lecture and that the DYE was useful in developing their personal interaction skills.

**Conclusion:**

DYE is a novel active learning model that can be incorporated into student courses as an alternative to traditional didactic lectures. Further development of the DYE technique, such as including supportive audio-visual resources, is necessary in order to increase student acceptance.

## 1 Introduction

Passing the torch of knowledge from the instructor to the learner has evolved throughout history. Traditional lectures required learners to humble themselves and become patient in order to acquire this inheritance (Gordon, 1997). Although instructors have occasionally adapted their teaching techniques to suit various learning styles, our generation has witnessed an overwhelming reversal of roles. More often than not, teachers are consistently seeking ways to adapt their teaching methods. They have become patient with the limited attention spans of learners and their desire for external stimuli (Oblinger, 2003). It has become imperative upon educators to continuously develop modern teaching methods to enhance learning, engage students, and use the most effective teaching techniques during their lectures (Al Shammari and Al Massaad, 2019).

Active learning techniques are commonly used in pharmacy education and are encouraged by the Accreditation Council for Pharmacy Education (ACPE) to develop problem-solving and critical-thinking skills (Allen et al., 2013, Alruthia et al., 2019, Gleason et al., 2011). These methods provide immediate feedback to both the instructor and the learner, enable students to take an active role in their learning, and assist students in making meaningful connections and associations (Michael, 2006, Prince, 2004). Although instructors are enthusiastic about implementing new approaches, designing or selecting new methods and evaluating their effectiveness, as compared to commonly used and/or published activities, can be challenging (Alruthia et al., 2019, Gleason et al., 2011).

Due to the COVID-19 pandemic, distance education is no longer optional. In fact, it is the only available choice to continue the education process during such circumstances. If properly applied, distance learning can serve as valuable solution in countries that have chosen to suspend physical attendance in schools and universities. Furthermore, there has never been more of an urgency to establish opportunities for supplemental e-learning (Tretter et al., 2020). The current situation requires innovation and cooperation between various healthcare disciplines to maintain rigorous standards of education and training for healthcare practitioners as well as students (Chick et al., 2020). Several efforts are being investigated to determine the best means of actively engaging learners remotely. In response to the recent changes in the delivery of medical education, the highly reputable *Medical Education* journal has released a new series called “Medical Education Adaptations” to encourage rapid dissemination of focused articles presenting innovative methods to deliver high-quality medical education during the current crisis (Eva and Anderson, 2020).

Gewin proposed five tips for moving teaching online during the COVID-19 pandemic (Gewin, 2020). These tips were (1) “Don’t convert your entire lecture to video ”, (2) “Don’t rely on live video”, (3) “Invite student engagement and feedback”, (4) “Check in with students often” and (5) “Identify and support struggling students”. The third tip advises educators to encourage student engagement and feedback because the most common mistake in the learning process is not considering your students' opinions.

Active learning strategies vary in type and the amount of effort required for effective execution. Popular examples in pharmacy education for larger classroom sizes include flipped classroom (FC) and team-based learning (TBL). The FC reverses the traditional didactic lecture with out-of-class learning activities and information reinforcement. TBL fosters an environment of learning and exploration within small student groups under the supervision and facilitation of the instructor. The utilization of pre- (individual or team readiness assurance tests, iRAT/tRAT) and end-of-class (EOC) quizzes are considered essential to TBL activities and have been associated with increasing exam scores of sub-optimally performing students, assisting students in identifying areas of improvement, and motivating students to prolong their studying time (Farland et al., 2013). Several past studies have shown the value of TBL pedagogy in the learning process. However, most TBL studies have been conducted in courses led by faculty with minimal examination of student-led learning courses (Bouw et al., 2015, Kolluru, 2012).

In the current article, we introduce a novel student-led active learning technique. Design Your Exam (DYE) is an active learning model that encourages students to prepare for the lecture prior to attending by reviewing the written material similar to FC (Wilson et al., 2019). However, instead of directing the student to read the material before attending the lecture and then assessing his/her understanding during the discussion, the student prepares the questions himself and tests his colleagues on it. Before the student reaches this stage, he/she must clearly understand the material before he/she can accurately test his/her colleagues on this information. Furthermore, this technique was hypothesized to improve students higher-level and critical thinking skills.

The objective of this study was to introduce the DYE activity into an Industrial Pharmacy course and compare its educational outcomes against instructor-designed EOC quizzes associated with the standard didactic method. Student scores, perceptions, and preferences for each teaching modality were compared to determine the most effective strategy.

## 2 Methods

### 2.1 Study design

The current study was conducted in the Industrial Pharmacy course in College of Pharmacy at King Saud University (Level 9, bachelor's degree, male campus). This course covers several important pharmaceutical industry processes and provides an overview of the various types of equipment utilized in these processes. By the completion of this course, students should gain the basic knowledge and skills that they need for employment in the production and/or quality control of pharmaceutical products. Examples of topics covered by the course include particle size reduction, particle size analysis, mixing, crystallization, tablet manufacturing, tablet coating, and capsule production.

### 2.2 Procedure

This course was delivered live. In our college, the traditional didactic lecture is the most common method utilized to deliver lectures. Since the DYE activity would be used for the first time in the Industrial Pharmacy course, the authors were concerned that applying this new activity to the entire course may cause confusion for the students initially. The lectures selected for the DYE method were closely related and considered to be more suitable to be delivered by the same method. Therefore, it was decided to conduct two-thirds of the course in the traditional format and one-third incorporating the DYE activity.

Accordingly, the first nine lectures of the course were delivered through the normal didactic method followed by instructor-designed EOC quizzes. Prior to the lecture, the instructor prepared 2–4 questions using Google Forms® that only covered the most important information from that day’s lecture (Lailaturrahmi et al., 2020). The quizzes were designed with question randomization and provided immediate grading/feedback. Ninety percent of the lecture time was dedicated to the instructor’s explanation. The remaining 10% of the lecture period was reserved for the EOC quizzes.

At the end of the lecture, the instructor sent the quiz link to the students, and each student took the test using his mobile phone after entering his student ID number. Students were instructed to answer questions without assistance from others and without using their notes (i.e. closed-book) (Leonard et al., 2012). Students learned independently and were provided with a complete explanation of the topics during the lectures; therefore, they independently answered the quizzes without any lecture information or collaboration. The students were given a maximum of five minutes to complete the quiz and were provided with their scores and any incorrect answers after completion. In addition, the instructor was able to view the students’ results immediately to assess their understanding of the lecture material. Student participation for every lecture was based upon whether they submitted the lecture-associated EOC quiz.

The subsequent four lectures of the course utilized the DYE active learning technique. Prior to each lecture, the instructor assigned reading material related to the lecture and divided it among the students into several short passages so that each student would read carefully and think critically about the part allocated to him (Dobson, 2008). After reading the material, the student prepared two questions about the part assigned to him in either a multiple choice or true/false question format. The student-designed questions were submitted to the instructor electronically with the correct answer identified. As an incentive to motivate the students to write accurate questions and to maintain confidentiality, extra credit was awarded to the student who prepared the most difficult question. Although each student was assigned a specific section of the reading material, he was still responsible for reading the entire reading assignment. After submission, the instructor reviewed the questions and revised them to prepare an electronic test to be given at the beginning of each lecture using Google Forms® consisting of 35–45 questions for each lecture. Typically, the instructor would choose the best of the two questions submitted by each student. Student participation was determined by whether or not the student submitted their questions by the specified deadline.

In order to maximize the accessibility and learning benefits of the activity, students were divided into groups of 5–6 members to compete with other groups in taking the open-book online exam (Lull and Mathews, 2016). In contrast to utilizing a fixed-grouping strategy (Chan et al., 2018), the groups were randomized each lecture by the instructor to encourage students to read all of the assigned material and not rely upon their colleagues. This strategy also minimized the opportunity for students to divide the reading material up among themselves in fixed groups prior to the pre-leture quizzes. Since the DYE was an active learning activity without instructor clarification and/or explanation initially, DYE quizzes consisted of approximately ten times the number of questions given on the EOC quizzes, and students primarily focused on their assigned reading material, a collaborative, open-book format was best suited for answering the DYE quiz questions. The group with the highest score was declared the winner. In the case that more than one group achieved the highest score, the group that completed the exam first would be declared the winner. Once the specified time for the exam was over, the instructor presented the test results and identified the student who submitted the most difficult question. At the end of the lecture or in subsequent lectures, the lecturer summarized the lecture material with an emphasis upon correcting the most common mistakes among students.

The results of the EOC or DYE lecture quizzes were not subjected to score comparison due to the major difference between testing methods in each technique; however, student comprehension was assessed during a midterm exam and a final exam.

The midterm exams were administered according to the pre-specified university schedule; therefore, it only covered 7 lectures of the EOC activity. On the other hand, the two activities overlapped in the final exam. The questions related to the lectures conducted with the instructor-designed EOC quizzes and the DYE activity were grouped and analyzed separately. In addition, differential student scores in the mid-semester and final exams were analyzed and categorized based on the implemented technique in each set of lectures. Each set of questions were analyzed and compared to determine whether there was a significant difference between student scores on either activity.

At the end of the course, a voluntary survey was sent to all students to solicit their feedback regarding both the instructor-designed EOC quizzes and DYE activities (Lull and Mathews, 2016). The survey was in the Arabic language and designed to evaluate student feedback anonymously. The survey was created using Google Forms® and the survey link was sent by email to the student group leader who forwarded the link to the other students. The survey consisted of three sections related to the EOC learning model, the DYE learning model, and general points and suggestions. There were a total of 20 questions; 12 were presented in a five-point Likert scale format to assess student perceptions of each technique. Furthermore, two multiple choice, one short-answer, and five open-answer questions were provided to identify the preferred learning model for the student, the existence of technical problems, suggestions, and advantages/disadvantages of each model.

### 2.3 Ethical considerations

The protocol for this research was reviewed and approved by the King Saud University Institutional Review Board (#E-21-5718).

### 2.4 Statistical analysis

SPSS 26 software was utilized to test the significance of the data. The percentage of students' attendance and activity participation were compared using independent *T*-test. Students’ scores were compared using paired *T*-test. *p* < 0.05 was denoted significant in the study.

## 3 Results

Fifty-two students were enrolled in the Industrial Pharmacy course in the fall 2019 semester. One student was prohibited from taking the final exam due to excessive absences that exceed the university attendance requirements. Accordingly, this student was excluded from the study; therefore, 51 students were included in the final statistical analysis.

The average attendance was approximately 78% for the instructor-designed EOC quizzes and 72% for the DYE activity (Table 1). Furthermore, student participation in the EOC and DYE activity was 75.0% and 78.9%, respectively. Mean exam performance from the midterm and final exams was 74.4% for the didactic method and 71.9% for the DYE activity (p = 0.092). The differential exam scores were generally comparable between the two techniques, but a higher percentage of students earned an A grade on the instructor-designed EOC quizzes. When comparing attendance, participation, and student scores, there was no significant difference between the two activities (Table 1).

**Table 1:** Students’ average attendance, participation and exam score.

Teaching technique	Average attendance (%)*	Participation (%)*	Student Score (100)
Instructor-designed end-of-class (EOC) quizzes	77.6 ± 11.7	75.0 ± 11.5	74.4 ± 15.2
Design Your Exam (DYE)	72.1 ± 3.3	78.9 ± 2.9	71.9 ± 14.8
Statistical test	Independent *t*-test	Independent *t*-test	Paired T-test
p Value	0.524	0.383	0.092

* Data are presented as percentage of total active class students (Mean ± SD).

Eighteen students completed the survey which represented a 39% response rate. Student responses were overwhelmingly positive for the EOC quizzes as shown in Table 2, Table 3. Approximately 90% of the students felt that the EOC quizzes helped them to understand the lecture material and nearly 80% and 90% responded that the EOC quizzes were useful/highly useful in helping them to understand the key information and keeping them focused during the lecture, respectively. In addition, 83.3% of the participants recommended that future offerings of the course incorporated EOC quizzes. Some examples of student self-reported advantages and disadvantages of the end-of-class quizzes are provided in Table 4.

**Table 2:** Student perception on understanding the lecture material and future preference.

Question	Learning Model	Excellent n (%)	Good n (%)	Acceptable n (%)	Weak n (%)	Very weak n (%)
How would you rate your understanding of the lecture material?	**Instructor-designed (EOC) quizzes**	9 (50)	7 (38.9)	2 (11.1)	0 (0)	0 (0)
	**Design Your Exam (DYE)**	0 (0)	2 (11.1)	8 (44.4)	6 (33.3)	2 (11.1)

**Question**	**Learning Model**	**I strongly support n (%)**	**I support n (%)**	**Neutral n (%)**	**I object n (%)**	**I strongly object n (%)**

Do you support incorporating these learning models in future course offerings?	**Instructor-designed (EOC) quizzes**	11 (61.1)	4 (22.2)	2 (11.1)	1 (5.6)	0 (0)
	**Design Your Exam (DYE)**	2 (11.1)	3 (16.7)	3 (16.7)	7 (38.9)	3 (16.7)

*The number under the rating column indicates the number of students who picked that option and the number within the parentheses is the corresponding percentage of students.

**Table 3:** Student perception on reading, focusing in the lecture and improving their interpersonal skills.

Activity	Questions	Very useful n (%)	Useful n (%)	Neutral n (%)	Not useful n (%)	Absolutely useless n (%)
Instructor-designed (EOC) quizzes	How useful was the activity in helping you to understand the most important information from the lecture?	12 (66.7)	2 (11.1)	3 (16.7)	1 (5.6)	0 (0)
	How useful was the activity in keeping you focused during the lecture?	9 (50)	7 (38.9)	1 (5.6)	1 (5.6)	0 (0)

Design Your Exam (DYE)	How useful was the activity in motivating you to read the lecture material before the class?	1 (5.6)	1 (5.6)	10 (55.6)	4 (22.2)	2 (11.1)
	How useful was the activity in keeping you focused during the lecture?	2 (11.1)	2 (11.1)	6 (33.3)	4 (22.2)	4 (22.2)
	How useful was the activity in developing your interpersonal skills (e.g. team work – decision-making)?	6 (33.3)	7 (38.9)	2 (11.1)	1 (5.6)	2 (11.1)

*The number under the rating column indicates the number of students who picked that option and the number within the parentheses is the corresponding percentage of students.

**Table 4:** Advantages and Disadvantages of the instructor-designed end-of-class quizzes.*

Advantages	Disadvantages
The activity enhances our comprehension of the information.	Students are accustomed to the (traditional) methods of learning, so it may cause difficulty for them
It motivates the student to focus during the lecture and gives them examples of the types of questions that will be asked on the exams.	The lecture material contains a lot of information that is not in the interest of the student.
It increases the student’s concentration during the lecture, even if he did not intend to do so because he knows that there is a quiz at the end of the lecture. If he answers it correctly then it will increase his grades in the subject.	It is not possible to focus for a full 45 min, which affects the quiz score even if the quiz questions are easy.
An exciting way for students to comprehend the information.	Internet coverage and poor network
(It helps us to) focus in the lecture and review what we have studied.	Not good if there are other tests (i.e. in other subjects)

* Only most important and relevant comments were presented.

The DYE activity was not as popular with the students (Table 2, Table 3). Equal proportions of the students (44.4%) stated that they either satisfactorily or poorly/very poorly understood the material when using the DYE model. The majority of students reported that the DYE was not useful/absolutely not useful or neutral in both motivating them to read the lecture materials before the class and motivating them to focus during the lecture. Although most students agreed that the DYE was useful in developing their personal interaction skills (72.2%), they did not recommend its use in the future (55.6%). Selected student quotes regarding the advantages and disadvantages of the DYE activity are provided in Table 5.

**Table 5:** Advantages and Disadvantages of the DYE.*

Advantages	Disadvantages
Establishing the concept and improving the skills of teamwork	You don't understand anything, except your (assigned reading) part.
It strengthens teamwork, research, and comprehension skills	Failure to fully understand the lecture material because my main focus while solving the DYE quiz is to search for the answer without comprehension or deep understanding of the key information
Enthusiasm and development of personal skills	Student understanding of the lecture material is a lot less than the first model

* Only most important and relevant comments were presented.

Students were also asked to compare the two models according to their preferences. When asked which model they preferred to be used in the Industrial Pharmacy course, 72.2% preferred the EOC quizzes. Approximately 16.7% recommended a combination between both models, and 5.6% chose either the DYE alone or another activity other than the current models (Table 2).

## 4 Discussion

In the current study, instructor-designed EOC quizzes and a student-designed assessment (DYE technique) were investigated for their potential enhancement of student engagement prior to and within the lecture. Both techniques had a positive influence upon student attendance, participation, and exam scores. Interestingly, most students preferred the instructor-designed assessments for understanding the lecture, but they confirmed the benefit of DYE for enhancing their communication and interaction skills.

Student participation was higher in the DYE activities than the EOC quizzes activities; however, the differences were not significant, and this could be due to the differences in the methods of determining participation. Regardless, student comments indicated that the EOC quizzes were successful in helping students to focus during the lecture periods. This may be due to the differences in delivery. The EOC quizzes were proceeded by a lecture with the understanding that there would be a quiz on the lecture material. In this study, a lecture followed the DYE activity; however, if a recorded lecture was made available to the students ahead of time, the students would probably focus on the lecture material to assist them in formulating questions.

Several previous studies showed positive educational outcomes of “EOC quizzes”. Chan et al. reported that pharmacokinetics workshops and post-workshop quizzes were instrumental in significantly increasing average final exam scores (Chan et al., 2018). Vinall and Kreys found in a single-blinded, randomized, controlled, crossover study that EOC quizzes encouraged student introspection related to their personal comprehension (Vinall and Kreys, 2020). Furthermore, Hennig et al. reported that feedback quizzes could enhance learning and performance and lead to improvements in student satisfaction with clinical pharmacokinetics courses (Hennig et al., 2019). In our study, the mean exam scores were 74.4% for the didactic method (with EOC quizzes) and 71.9% for the DYE activity (p = 0.092). Differences in student mean exam scores related to each activity were not significant. These results are in agreement with previous systematic reviews of the pharmacy, medical, and nursing literature which have shown that the FC instructional design has the same effect on student performance as didactic lectures (Wilson et al., 2019). A meta-analysis of the FC in pharmacy education did show a small, positive increase in performance, though this was not statistically significant. The non-significant differences in student attendance, participation, and exam score results indicate that the DYE activity is a novel active learning activity and can be incorporated into student courses as an alternative to the standard didactic lectures. Furthermore, these results indicate that the DYE is non-inferior to current active learning methods such as the EOC quizzes.

Student perceptions of the EOC quizzes were more favorable than for the DYE activities. In our study, 72.2% of students preferred the didactic with EOC quizzes method, 5.6% preferred the DYE activity and 16.7% preferred a mix of both models. This data can be correlated with a study by (Wilson et al., 2019) which found that 5.7% of students preferred the flipped method alone and 47.5% preferred a mix of the didactic and flipped methods. The decreased student preference for DYE might be due to their familiarity with the didactic method and the desire for instructor-led reinforcement of content (Giuliano and Moser, 2016, Khanova et al., 2015).

On the other hand, most students agreed that the DYE was useful in developing their personal interaction skills such as teamwork and decision-making. These results are in agreement with previous studies which showed that students positively perceived student-led TBL activities as a proactive type of peer-to-peer teaching (Bouw et al., 2015). The students perceived that peer-led activities improved their interpersonal communication, presentation, teamwork, leadership, and evaluation skills, which are all essential for future pharmacy graduates to function as a cohesive team in a wide range of healthcare fields (Valler-Jones, 2014)

Several studies highlighted the importance of considering the time required for pre-course preparation when designing student-led pre-class activities (Wilson et al., 2019). In the current study, the assigned reading materials were planned to be short and concise for each student. This step was undertaken to minimize cheating. However, the survey comments indicated that the students focused specifically upon their assigned reading sections and neglected reading the entire assigned lecture materials in the DYE activity. This might be one the reasons for the low student perception of DYE activity in terms of understanding the scientific information of the lecture.

Although the students benefitted from the student-designed DYE assessments, incentives such as requiring students to work in teams when submitting their questions and/or further explanation of the material such as recorded lectures, instructional videos, or interactive e-lectures may enhance student comprehension.

Several limitations should be discussed. Due to the low response rate of the students and limited number of participants, the results of the study may not be generalizable. Future studies should include larger samples, multiple class cohorts, or students from multiple universities. In addition, all questions used in the exam comparison were multiple choice. It may be helpful to compare student performance using other assessment strategies, such as short-answer questions, which may be better suited to evaluate critical thinking skills. In addition, providing the students with more explanation of the material prior to submitting their DYE questions may encourage students to review the entire assigned material, design high-quality questions, and facilitate better student comprehension. Finally, the acquisition of advanced software, such as a plagiarism checker, may prevent the duplication of student-designed questions.

There are several potential areas for future study such as assessing the impact of student-led activities such as DYE on student knowledge retention. Further studies to select content areas most suited for DYE would be helpful. A study on student burden and the optimum pre-class preparation time across the curriculum could help pharmacy faculty in implementing student-centered learning activities. More robust studies are needed to shed light on the connection between student-led activities and improvements in learning, understanding, and satisfaction. Although this study was conducted prior to the COVID-19 crisis and the lectures were delivered live, it provides a platform for improving e-learning and encourages students to play an active role in distance learning. This pilot study suggests that students may perceive student-led activities positively and that further studies are warranted.

## 5 Conclusion

Student-led active learning activities encourage students to play an active role in the classroom. Currently, instructors must explore new active learning techniques that can enhance students' engagement particularly within in the challenging distance learning environment. The DYE activity is a novel method of active learning that can be easily incorporated into the classroom setting. Further development of the DYE technique, such as including supportive audio-visual resources, is necessary to increase student acceptance.

## Acknowledgement

The authors extend their appreciation to the Deanship of Scientific Research at King Saud University for funding this work through research group no. (RG-1441-414).

## Declaration of Competing Interest

The authors declare that they have no known competing financial interests or personal relationships that could have appeared to influence the work reported in this paper.
